# Evaluation of Several Satellite-Based Soil Moisture Products in the Continental US

**DOI:** 10.3390/s22249977

**Published:** 2022-12-18

**Authors:** Shouming Feng, Xinyi Huang, Shuaishuai Zhao, Zhihao Qin, Jinlong Fan, Shuhe Zhao

**Affiliations:** 1 Jiangsu Center for Collaborative Innovation in Geographical Information Resource Development and Application, School of Geography and Ocean Science, Nanjing University, Nanjing 210023, China; 2 Yellow River Lijin Bureau, Yellow River Conservancy Commission, Lijin 257400, China; 3 MOA Key Laboratory of Agricultural Remote Sensing, Institute of Agro-Resources and Regional Planning, Chinese Academy of Agricultural Sciences, Beijing 100081, China; 4 National Satellite Meteorological Center, Beijing 100081, China

**Keywords:** satellite-based soil moisture products, evaluation, continental US, data quality, in situ networks

## Abstract

Satellite-based soil moisture products are suitable for large-scale regional monitoring due to the accessibility. Five soil moisture products including SMAP, ESA CCI, and AMSR2 (ascending, descending, and average) were selected in the continental United States (US) from 2016 to 2021. To evaluate the performance of the products and assess their applicability, ISMN (International Soil Moisture Network) data were used as the in situ measurement. PBIAS (Percentage of BIAS), R (Pearson correlation coefficient), RMSE (Root Mean Square Error), ubRMSE (unbiased RMSE), MAE (Mean Absolute Error), and MBE (Mean Bias Error) were selected for evaluation. The performance of five products over six observation networks and various land cover types was compared, and the differences were analyzed at monthly, seasonal, and annual scales. The results show that SMAP had the smallest deviation with the ISMN data because PBIAS was around −0.13, and MBE was around −0.02 m^3^/m^3^. ESA CCI performed the best in almost all aspects; its R reached around 0.7, and RMSE was only around 0.07 m^3^/m^3^ at the three time scales. The performance of the AMSR2 products varied greatly across the time scales, and increasing errors and deviations showed from 2016 to 2020. The PBO_H2O and USCRN networks could reflect soil moisture characteristics in the continental US, while iRON performed poorly. The evaluation of the networks was closely related to spatial distributions. All products performed better over grasslands and shrublands with R, which was greater than 0.52, and ubRMSE was around 0.1 m^3^/m^3^, while products performed worse over forests, where PBIAS was less than −0.62, and RMSE was greater than 0.2 m^3^/m^3^, except for ESA CCI. From the boxplot, SMAP was close to the ISMN data with differences less than 0.004 m^3^/m^3^ between the median and lower quartiles.

## 1. Introduction

Soil moisture is a relative variable in a certain region, which is expressed as the percentage of soil moisture content per unit volume to the net weight of the soil sample [1]. As an important element in the terrestrial surface system [2], soil moisture affects the ecological environment, water resource utilization, agricultural irrigation, and other processes, reflecting the interaction among the pedosphere, hydrosphere, atmosphere, and biosphere [3,4,5]. Soil moisture is closely related to soil conditions, water resource distribution, survival, and behavior of organisms. It is also an important variable in the simulation analysis of hydrology during a specific watershed. Satellite-based remote sensing is still the mainstay of soil moisture estimation [6], due to the accessibility of global-scale measurements at a continuous spatiotemporal resolution [7]

Airborne and spaceborne satellite data for earth observation can be retrieved to obtain soil moisture products with a high temporal resolution. Based on active and passive remote sensing, research institutions have released various soil moisture products (such as SMAP, ESA CCI, and AMSR2). Despite the limitations of partial coverage and inversion results, data are available in most regions of the world. To improve data quality, more and more reconstruction and filling algorithms have been proposed and applied [8]. The evaluation helps to improve satellite-based remote sensing products and their retrieval algorithms [9], and the evaluation with statistical indicators [10] is of great significance for data applications in climate change and geographical environmental differentiation.

Satellite-based products are based on visible/infrared and microwave bands [11] and are often downscaled to medium spatial resolution for practical production applications [12]. These products have a good retrieval effect of soil moisture (0–5 cm) on the Earth’s surface, which is more suitable for analysis [13]. Existing studies combined several widely used products to evaluate their characteristics in different regions and explore their connection with the local environments. The regional differentiation is usually obvious.

Due to its continuity, SMAP has attracted more attention and is more widely used. Experiments based on several sites have shown that SMAP performed well in North America [14]. SMAP can also be used to compare the performance of different observation networks, but the temporal variation of soil moisture cannot be detected [15]. Some studies evaluated the performance of the ASCAT product in agricultural areas of the Southern United States and explored its ability to characterize drought by calculating relative soil moisture and the correlation coefficient [13]. Some studies compared the performance of SMAP, SMOS, and ASCAT comprehensively and evaluated three kinds of products together [16]. The observation sites were mostly concentrated in the continental US.

Due to the advantage of a long time series, in situ observation data are often used to evaluate the performance of soil moisture products between different sites [17]. However, a systematic evaluation over the observation networks is rare. The performance over different networks receives less attention, as stations with continuous observation are evaluated more often. Different stations in the observation networks share certain similarities, and evaluating the performance of them helpful to study the impact of geographical distribution.

Satellite-based soil moisture products could monitor drought and assess its evolution, which is essential to regions that lack stations [18]. Soil moisture could be converted into a drought index, which is used to classify and quantify the drought degree [19]. However, the evaluation from different time scales tended to be ignored in previous studies. When evaluating at different time scales, multiscale comparisons can reduce the time-lag effect between soil moisture and climate characteristics. Different time scales of soil moisture can capture various drought characteristics [20] and especially connect to the time scale of the drought index [21]. The assessment at different time scales over in situ observations is important for providing continuity and spatial mapping during drought monitoring [22].

Soil moisture products are of various types according to the attributes, such as active, passive, and combined remote sensing retrieval methods and a time range of ascending and descending. Comparing soil moisture products with long-term observation data can comprehensively evaluate the performance of various types [23]. Based on the in situ observational data of ISMN in the continental US from 2016 to 2021, this study selected PBIAS, R, RMSE, ubRMSE, MAE, and MBE to evaluate five products (SMAP, ESA CCI, and three types of AMSR2). The performance of different observation networks and the relationship with their spatial distribution was compared. The evaluation of soil moisture products was conducted at monthly, seasonal, and annual scales, with differences worth further analyzing. Four land cover types were divided to assess the performance of five products. The boxplot was used to analyze the data quality of the soil moisture products, which was related to the data validity and a reflection of retrieval algorithms.

## 2. Materials and Methods

### 2.1. Study Area and Data

In order to evaluate the performance of different soil moisture products in the continental US, in situ-measured data from observation stations and satellite remote sensing products were selected.

The International Soil Moisture Network (ISMN) is a comprehensive network applied in soil moisture verification with land surface and hydrology studies [24]. Data collecting networks share data with the ISMN voluntarily [25], supporting various depths of soil moisture measurements. The most commonly used techniques for systematic in situ sampling are based on the contrasting dielectric properties of soil and water [26], and global positioning system (GPS) sensors are also used for measuring soil moisture. Various quality control methods for soil moisture measurements are applied for the ISMN, which can be divided into geophysical dynamic range verification, geophysical consistency, and spectrum-based methods [27]. Stations of the ISMN are distributed in many regions, with a dense observation distribution in Europe, the United States, and Central Asia. Important areas of ecological protection also have numerous stations, such as wetlands and forests.

The Soil Moisture Active Passive (SMAP) product, which is provided by the National Snow and Ice Data Center, is designated by the National Aeronautics and Space Administration (NASA). This Level-2 (L2) soil moisture product is geophysically retrieved by both the Soil Moisture Active Passive radiometer and the Sentinel-1 radar and is resampled to a fixed Equal-Area Scalable Earth Grid [28]. SMAP L-band brightness temperatures and Copernicus Sentinel-1 C-band backscatter coefficients are used to derive soil moisture data, taking advantage of the much finer spatial resolution of 3 km and 1 km. The product was available from April 2015 to the current date with a temporal resolution of 12 days. The product with 3 km resolution was used because it had undergone validation.

The Climate Change Initiative Surface Soil Moisture Product (CCI) provided by the European Space Agency (ESA) is of various types [29]. All types of gridded soil moisture products are retrieved from radiometrically calibrated backscatter or brightness temperature measurements [30]. By integrating data from multiple satellite sensors, ACTIVE, PASSIVE, and the COMBINED products are produced by ESA [31]. The COMBINED product was used during this study, which harmonized ACTIVE and PASSIVE datasets by following a decision tree and performed better in both densely and sparsely vegetated areas [30] and is more suitable to drive hydrological studies [32]. The latest version of dataset (v07.1) improves the model parametrization and used a bias correction method in harmonizing datasets. ESA CCI is suitable for a wide range of soil moisture assessments, with a spatial resolution of 0.25° and a daily temporal resolution. The datasets cover the period from 1991 to 2021.

Based on research from VU University Amsterdam and NASA, the Land Parameter Retrieval Model (LPRM) is developed and applied to the Advanced Microwave Scanning Radiometer 2 (AMSR2). AMSR2 soil moisture products are retrieved from LPRM, which is based on a forward radiative transfer model [33] with improved calibration. AMSR2 products have better accuracy than previous series of AMSR [34]. Many microwave frequencies and different polarizations for the sensors can be applied to derive the soil moisture datasets [33]. The microwave radiometer scans the observations twice a day; the equator overpass times are at 13:30 p.m. local time for the ascending path and at 1:30 a.m. local time for the descending path [23,35]. The datasets cover the period from 2015 to the current date with a daily temporal resolution and a 10 km spatial resolution. Information about products is listed in Table 1.

The Terra and Aqua Combined Moderate Resolution Imaging Spectroradiometer (MODIS) Land Cover Type (MCD12 Q1) product [36] was used to evaluate the performance of five products for different land cover types, which was closely related to the soil conditions. The product contains five classification schemes and land cover property assessments. The land cover types were refined after supervised classifications, which incorporated prior knowledge and ancillary information.

**Figure 1:**
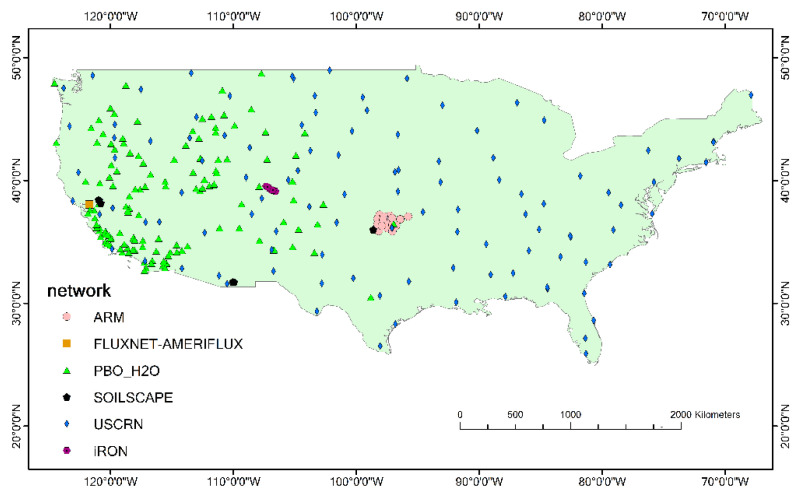
ISMN observation network distribution in the continental US.

Data of the ISMN observation networks in the continental US from 2016 to 2021 were selected in this study. Six ISMN observation networks with the names of ARM, Fluxnet-Ameriflux, PBO_H2O, SOILSCAPE, USCRN, and iRON distributed 17, 1, 135, 88, 112, and 9 observation stations, respectively. SMAP and ESA CCI products are available over the ARM, PBO_H2O, SOILSCAPE, USCRN, and iRON observation networks, while AMSR2 is available over all observation networks. The distribution of the observation networks and its stations is shown in Figure 1.

From the distribution of the observation networks, ARM is concentrated on the border of Kansas and Oklahoma. PBO_H2O is distributed in the western part of the continental US and is more concentrated in the southwest coast. The USCRN is distributed uniformly in the eastern part of the continental US. FLUXNET-AMERIFLUX is distributed in central California, while IRON is concentrated in central Colorado. SOILSCAPE is located in three dense locations: central California, southern Arizona off the coast, and western Oklahoma. To compare the dominant types over the ISMN networks, each station with land cover types that appeared the most were considered to be correct. The statistic of the results is shown in Table 2. Grasslands and shrublands were the dominant type over the ARM and PBO_H2O networks and were still the main part over the SOILSCAPE and USCRN networks. Savannas were the main type over the iRON network, to be the second main type of land cover over the SOILSCAPE and USCRN networks. In addition, wetlands were the only land cover type over the FLUXNET-AMERIFLUX network, because only one station existed.

### 2.2. Method

Considering that various satellite-based products are influenced by different orbits and revisiting periods of satellites, observation methods have great impacts on the products. Therefore, various soil moisture products cannot be directly compared, but can be compared with in situ-measured data [26]. Each soil moisture product was compared with ISMN data. Some 0–5 cm depth data of ISMN were retained with a good quality flag, which were averaged and synthesized by month. The nearest interpolation method was utilized to resample ESACCI and ASMR2 products to a spatial resolution of 3 km, the same as that of SMAP for evaluation [37,38]. For each soil moisture product, data with the same time and space location as ISMN were retained through sampling, according to the locations of ISMN observation stations, which were represented as points. Average values of AMSR2 products were calculated based on ascending and descending data. To verify the performance, three different AMSR2 product types were compared.

A land cover product derived from the International Geosphere-Biosphere Programme (IGBP) was used for evaluation. Based on the 17 types listed in the IGBP land cover product, land cover types used in the study were divided into four categories in order to simplify the evaluation [39], forests, grasslands and shrublands, savannas, and barren and sparse vegetation. Water and permanent wetlands were not considered, which took up less than 1% of total stations. Detailed classes corresponding to IGBP are listed in Appendix A. Distinct land cover types were identified at each station of various years through sampling, which were consistent with the MCD12Q1 product. Soil moisture products were compared at various land cover types in the corresponding year to make sure that changes in land cover types could be appropriately compared.

PBIAS (Percentage of BIAS), R (Pearson correlation coefficient), RMSE (Root Mean Square Error), ubRMSE (unbiased RMSE), MAE (Mean Absolute Error), and MBE (Mean Bias Error) were used to evaluate [40,41]. The expressions were as follows:

(1) $$\mathrm{PBIAS}=\frac{\sum \left(O_{i}-P_{i}\right)}{\sum O_{i}}\;$$

(2) $$R=\frac{\sum \left(O_{i}-\bar{O}\right)\left(P_{i}-\bar{P}\right)}{\sqrt{\sum {\left(O_{i}-\bar{O}\right)}^{2}}\sqrt{\sum {\left(P_{i}-\bar{P}\right)}^{2}}}$$

(3) $$\mathrm{RMSE}=\frac{1}{n}\sqrt{\sum {\left(O_{i}-P_{i}\right)}^{2}}$$

(4) $$\mathrm{ubRMSE}=\sqrt{\frac{1}{n}\sum {\left[\left(O_{i}-\bar{O}\right)-\left(P_{i}-\bar{P}\right)\right]}^{2}}$$

(5) $$\mathrm{MAE}=\frac{1}{n}\sum \left|O_{i}-P_{i}\right|$$

(6) $$\mathrm{MBE}=\frac{1}{n}\sum \left(O_{i}-P_{i}\right)$$

where $O_{i}$ represents data of the ISMN; $P_{i}$ represents the value of soil moisture products at the corresponding position, and *n* is the amount of effective data. PBIAS and MBE compare the average trend between ISMN data and products by measuring the deviation [42], indicating that products overestimate/underestimate ISMN data. Positive values of PBIAS and MBE indicate underestimation, while negative values indicate overestimation. R represents the correlation between ISMN data and products, and the correlation increased with the absolute value. RMSE, ubRMSE, and MAE represent the error between ISMN data and products, and errors increased with the value.

At monthly scale, the performance over the observation networks was assessed. Detailed comparative analyses were carried out at monthly, seasonal, and annual scales according to the average values. Seasons were divided into spring (March–May), summer (June–August), autumn (September–November), and winter (December–February) [43]. Overall performance was compared by scatter plots and boxplots. Statistics included quartiles that were calculated, and outliers could reflect the difference of data quality control.

## 3. Results

### 3.1. Comparative Analysis over Observation Networks

**Figure 2:**
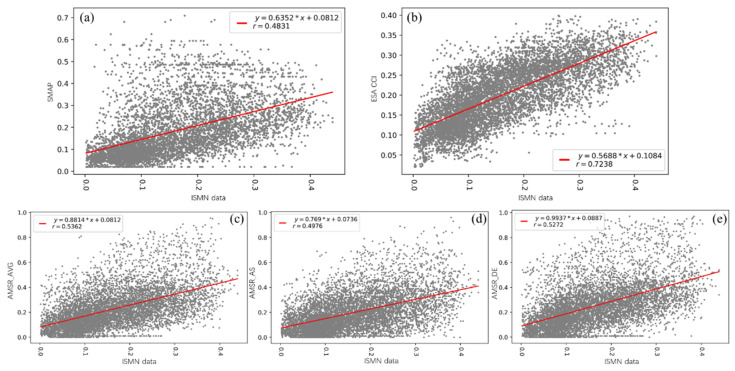
Scatter plots between several products and ISMN data ((**a**) SMAP, (**b**) ESA CCI, (**c**) AMSR2 average product (AMSR_AVG), (**d**) AMSR2 ascending product (AMSR_AS), and (**e**) AMSR2 descending product (AMSR_DE)).

The correlations among several products and ISMN data were objectively shown by scatter plots (Figure 2), and six statistical indicators were calculated.

Considering the differences in the number of stations available and the data availability over the six observation networks, this study evaluated the performance of five products according to the classification and distribution of the observation networks. The AMSR2 products were available at six observation networks, while the SMAP and ESA CCI products were not available at the FLUXNET-AMERIFLUX observation network.

In terms of PBIAS in Table 3, SMAP underestimated the ARM, PRO, and iRON observation networks and overestimated the SOILSCAPE and USCRN observation networks. The ESA CCI and AMSR2 products overestimated the entire observation networks. In terms of R, the five products showed a good correlation with ISMN data over the PRO and SOILSCAPE observation networks; ESA CCI showed a good correlation with ISMN data over the ARM and USCRN observation networks, and all products performed poorly over the iRON observation network. ESA CCI performed the best in both RMSE and MAE, while SMAP generally performed better in MBE. The average values of AMSR2 performed well in multiple indicators over Fluxnet-Ameriflux. The products with the best performance of each indicator are listed in Table 4.

### 3.2. Overall and Detailed Comparative Analysis at Different Time Scales

**Figure 3:**
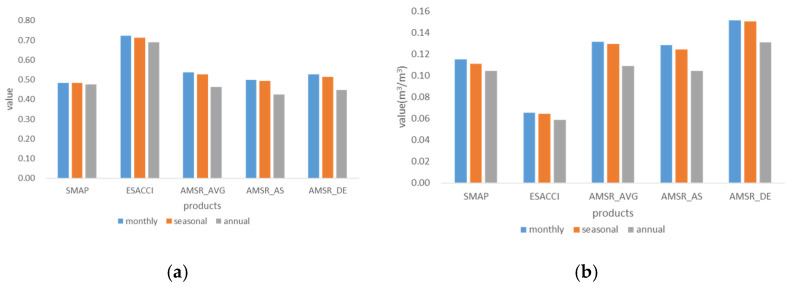
Overall performance of five products at different time scales. (**a**) performance of R, *p*-value < 0.0001; (**b**) performance of ubRMSE.

Slight differences showed among the monthly, seasonal, and annual scales when we evaluated the overall performance of the five products. Even so, some trends are illustrated in Figure 3. ESA CCI showed the highest correlation around 0.7 and the minimum error around 0.06 m^3^/m^3^. Five products performed differently with the time scales varied. R generally increased from the annual to seasonal scales of all products, and three types of AMSR2 increased by 0.066 on average. R increased slightly from the seasonal to the monthly scales, except SMAP. From the annual to seasonal to monthly scales, ubRMSE increased in all products and showed increased errors.

**Figure 4:**
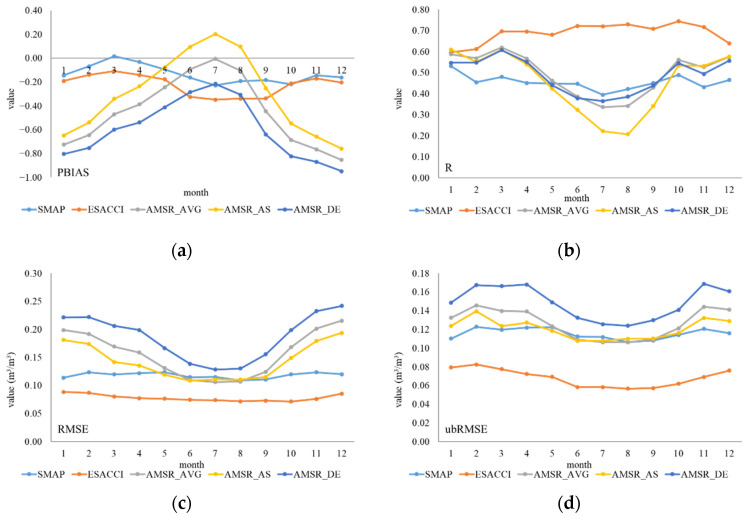

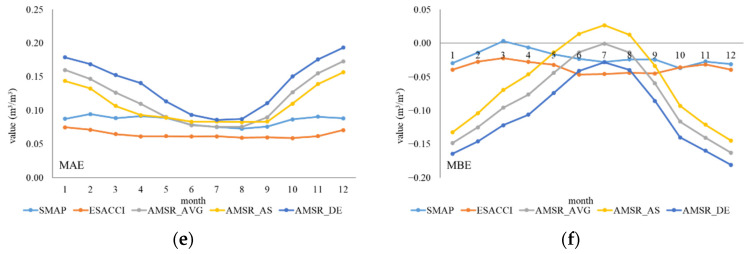
Performance of six indicators of five products at different months. (**a**) PBIAS; (**b**) R (*p*-value < 0.0001); (**c**) RMSE; (**d**) ubRMSE; (**e**) MAE; and (**f**) MBE.

The detailed performance of different months, seasons, and years was evaluated. The individual performance of the six indicators was calculated for each month and is shown in Figure 4. In terms of PBIAS and MBE, five products overestimated ISMN data in most months, except AMSR_AS from June to August and SMAP in March. Generally, SMAP showed the smallest deviation with ISMN data. ESA CCI showed the highest correlation with ISMN data in almost all months. The R of AMSR_AS showed large fluctuations from 0.2 to 0.6. In terms of RMSE, ubRMSE, MAE, and ESA CCI showed the minimum error, while AMSR_DE showed the maximum error with ISMN data. Three products of AMSR2 indicated similar trends and displayed similar ranks except for R. For AMSR2 products, the errors and deviations decreased gradually from January to June and increased gradually from August to December. However, R showed the opposite features. Although the errors and deviations were the smallest between June and August, the correlation with ISMN data was the lowest of the year, which meant a poor performance.

**Figure 5:**
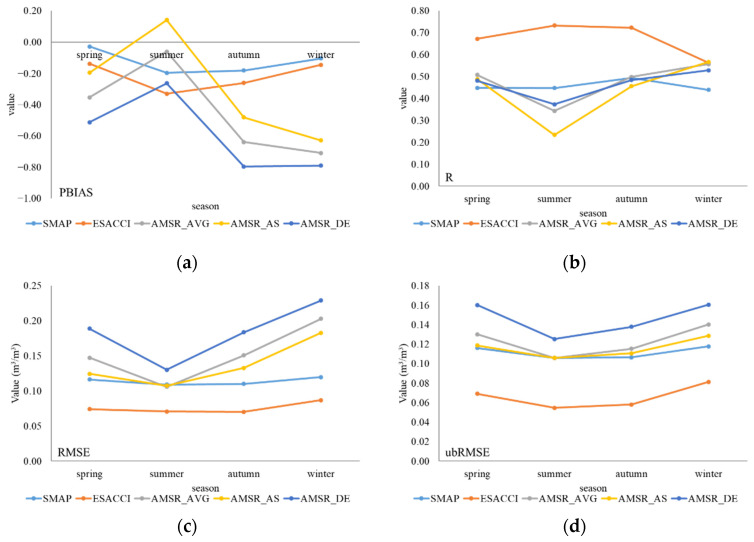

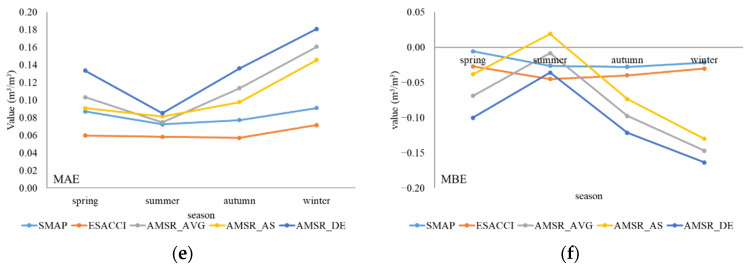
Performance of six indicators of five products at different seasons. (**a**) PBIAS; (**b**) R (*p*-value < 0.0001); (**c**) RMSE; (**d**) ubRMSE; (**e**) MAE; and (**f**) MBE.

The performance of different seasons shown in Figure 5 reflects the influence of climate on seasonal changes. In terms of PBIAS and MBE, all products showed minimum error in summer; three types of AMSR2 products showed obvious error in autumn and winter. In terms of R, ESA CCI performed better but dropped evidently in winter. Three types of AMSR2 products performed the best in winter and performed worst in summer. In terms of RMSE, ubRMSE, MAE, and ESA CCI showed the minimum error, while AMSR_DE showed the maximum error with ISMN data. AMSR2 products showed the least errors and deviations in summer, which was consistent with that of the monthly scale. However, both errors and deviations increased from summer to winter, while the correlation also showed opposite features. The differences and relativities of AMSR2 products seemed incongruous.

**Figure 6:**
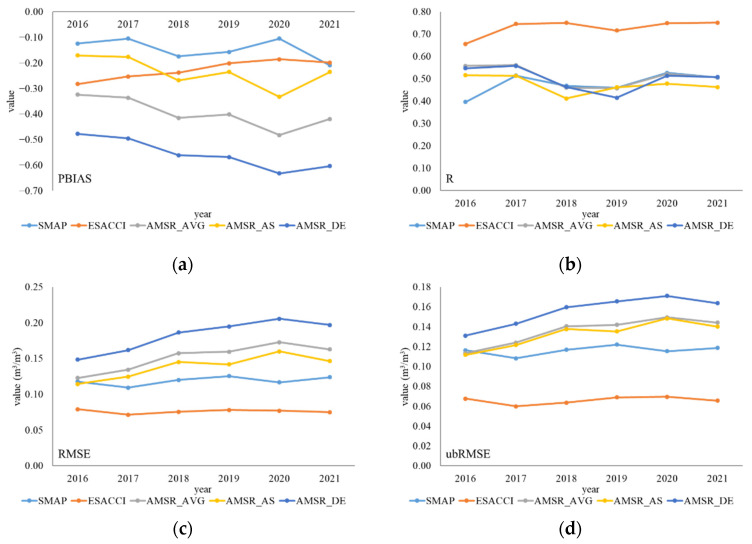

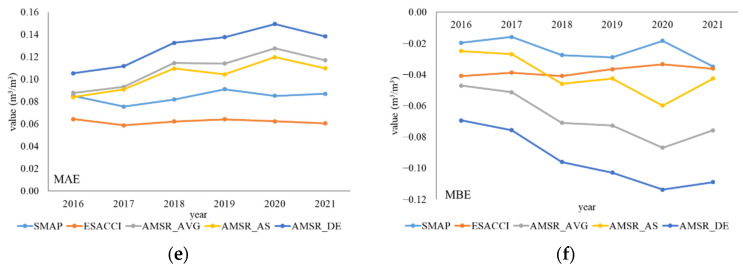
Performance of six indicators of five products at different years. (**a**) PBIAS; (**b**) R (*p*-value < 0.0001); (**c**) RMSE; (**d**) ubRMSE; (**e**) MAE; and (**f**) MBE.

From the perspective of the annual scale, the performance of several products varied slightly among the years (Figure 6). Products overestimated ISMN data across all years, while ESA CCI kept the best correlation with ISMN data. SMAP showed the smallest deviation with ISMN data, while AMSR_DE showed the highest deviation, which was consistent with the overall performance results. The results are consistent with those at the monthly and seasonal scales in terms of RMSE, ubRMSE, and MAE; ESA CCI showed the minimum error, while AMSR_DE showed the maximum error with ISMN data. PBIAS and MBE of AMSR2 products decreased, while RMSE, ubRMSE, and MAE increased from 2016 to 2020, reflecting the increasing errors and deviations during the period. A worse performance was inferred from the AMSR2 products year by year, only to improve in 2021.

### 3.3. Performance over Land Cover Types at Monthly Scales

**Figure 7:**
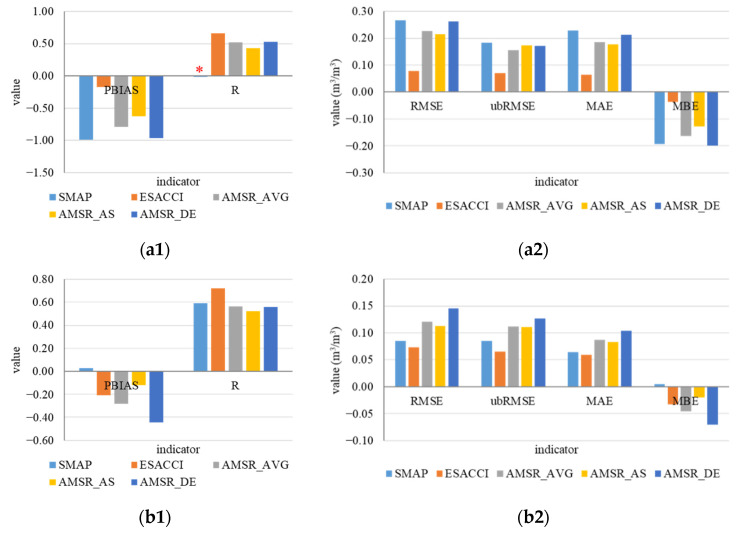

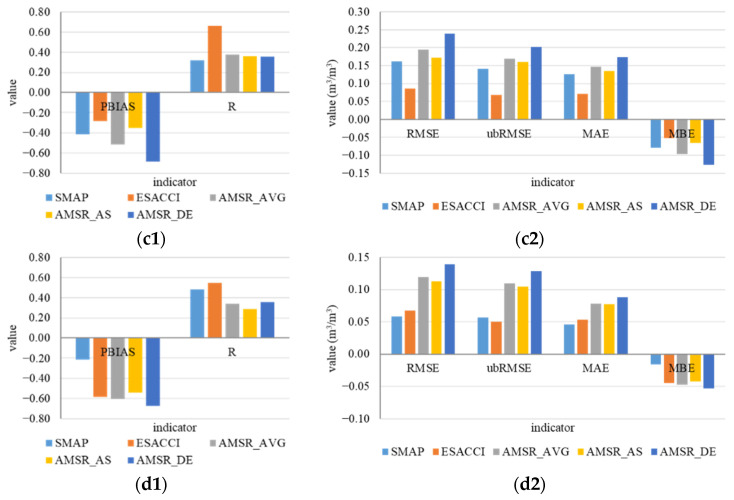
Performance of land cover types at monthly scale (for R, *p*-value < 0.0001; except R of SMAP in subplot (**a1**) marked with red asterisk, its *p*-value was 0.78 with no significance). (**a1**,**a2**): forests; (**b1**,**b2**): grasslands and shrublands; (**c1**,**c2**): savannas; and (**d1**,**d2**): barren and sparse vegetation.

The performance over different land cover types varied greatly on indicators (Figure 7). Over forests, SMAP performed poorly with the highest deviation and error, and no correlation showed with ISMN data. Over grasslands and shrublands, SMAP performed well with the lowest PBIAS and MBE and was the only product that did not show overestimation. The R of SMAP reached around 0.6 and was only inferior to ESA CCI. AMSR_DE showed the highest deviation and error although R reached 0.55. Over savannas, SMAP and AMSR2 performed moderately and showed similarities. Over barren and sparse vegetation areas, the AMSR2 products performed worse; ESACCI showed high deviation with low error. ESA CCI performed almost best on all indicators. Compared with four land cover types, five products performed better over grasslands and shrublands, with higher correlation and lower deviation and error with ISMN data. Greater deviation and error showed over forests, and the results showed higher overestimation with ISMN data.

### 3.4. Data Quality Analysis

**Figure 8:**
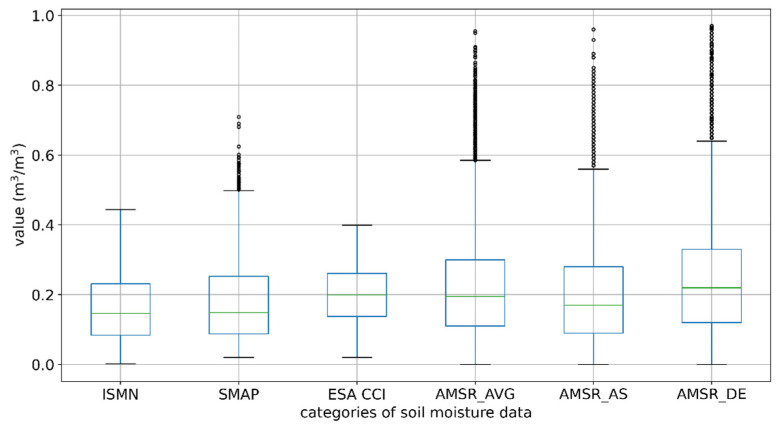
Boxplot of ISMN data and five products.

The study compared statistical values of five products and obtained the maximum ISMN value of 0.4438 m^3^/m^3^ from 2016 to 2021. However, there were 325, 0, 685, 512, and 910 numbers of data larger than 0.4438 m^3^/m^3^, corresponding with the SMAP, ESA CCI, AMSR2_AVG, AMSR2_AS, and AMSR2_DE products, which reflected the differences in data quality control. In order to visually display the differences, the upper quartile Q3 and the lower quartile Q1 of each product were calculated, thereby obtaining the interquartile distance IQR = Q3–Q1. With Q3 + 1.5 IQR as the upper boundary and Q1-1.5IQR as the lower boundary, the outlier data were screened out, and a boxplot was drawn in Figure 8. The statistical results are shown in Table 5.

According to the number and distribution of the outliers, the data quality control of ESA CCI was better. SMAP was close to the data of the ISMN according to similar quartiles and other statistics, while the overall range fluctuation of AMSR2 was the largest. All the quartiles of the products tended to be higher than ISMN data, confirming the overestimation of ISMN data. ESA CCI and SMAP both set a minimum value for soil moisture of 0.02 m^3^/m^3^ in the process of data processing, which had a certain effect on the exclusion of outliers.

## 4. Discussion

### 4.1. Applicability of Soil Moisture Products over Different Observation Networks

In different scenarios, it is of significance to select suitable soil moisture products according to the performance. The performance over the observation networks can help us to consider the application of products, as well as working with observation network data properly. From the perspective of R, all products were suitable for the application over the PBO_H2O, SOILSCAPE, and USCRN networks. Only the AMSR2 products were available over the FLUXNET-AMERIFLUX network, and a good performance indicated the potential effective use of this network. The deviation between the iRON network and products was large, and the correlation was weak. Therefore, how to effectively analyze the data of the iRON network and consider its usage are worth further discussion.

Considering the distribution of the observation networks, a poor performance was comprehensible in areas where iRON was concentrated. The soil moisture data were similar in terms of the impact of low spatial resolution, not to mention the lack of observatories and available data. Only one observation station of the FLUXNET-AMERIFLUX network ensured data consistency and performed well, which avoided the influence caused by similar values of the near stations. The SOILSCAPE network was concentrated in three areas, including 61 sites in central California, 15 sites in southern Arizona, and 12 sites in western Oklahoma. Therefore, the performance of the SOILSCAPE network was significantly affected by its location in central California. The good connection between the observation networks and the soil moisture products indicated that satellite-based products could be well applied to central California, which was consistent with the good performance of the FLUXNET-AMERIFLUX network in central California.

The PBO_H2O and USCRN networks evenly covered the western and eastern parts of the continental US, which effectively reflected the spatial differences of ISMN data and soil moisture products. Both in terms of performance and data coverage, the PBO_H2O and USCRN networks were more suitable for large-scale assessments and the representation of soil moisture distribution in the continental US.

### 4.2. Performance Comparison of Various Soil Moisture Products

In terms of SMAP, the overall deviations with ISMN data at different time scales were minimal. The performance of six indicators at monthly and seasonal scales did not differ significantly between months, indicating a smooth application of each month or of the months. The statistics were close to ISMN data, indicating that the overall distribution of SMAP was consistent with ISMN data.

In terms of ESA CCI, it showed better performance at three time scales. The R of ESA CCI was the highest around 0.7, and RMSE, ubRMSE, and MAE also indicated the minimum error. PBIAS and MBE were only slightly inferior to SMAP representing the low deviation. No obvious fluctuation showed at three time scales, which reflected the good stability of the data. ESA CCI almost performed the best over the observation networks at the monthly scale, which was more suitable for data analysis and studies under general circumstances. 

In terms of the AMSR2 products, the performance varied dramatically across the time scales. The applicability and improvement need further discussion, because their performance of correlations and differences showed contrasts, which was mentioned in the results. At different time scales, the errors and deviations of AMSR_AS were better than AMSR_AVG and AMSR_DE, but the results of the correlation were reversed. AMSR_AVG tended to perform better over different observation networks, especially over the FLUXNET-AMERIFLUX network. According to the boxplot, there were many outliers in the three AMSR2 products, and AMSR_AS was generally closer to ISMN data.

In general, the ESA CCI product seem to performed better for the whole evaluation [37,38,44,45], which was consistent with the results in this study. The ESA CCI product was affected least by the land surface temperature, vegetation water content, soil clay content, and other factors [46]. In addition, the in situ data of the ISMN were used for the validation of ESA CCI [26], compared to the other distributed observation networks.

The SMAP product also showed a good performance during existing studies, especially in the context of the soil moisture anomaly detection [45]. In other studies, the SMAP product had reached the highest correlation with some networks in the US [9]. However, the time period from 2015 to 2017 was shorter compared to the other products when the evaluation was conducted [9]. The ESA CCI product was updated to the latest version and was evaluated in this study, and its improvement showed a better performance, which might be the possible reason for the discrepancy with some results.

The AMSR2 products performed poorly and were reported with greater amplitude than the in situ measurements [37,38]. Like our results, ubRMSE of the AMSR2 products was much higher than other products over the three time scales. At monthly and seasonal scales, poor correlations showed in the summer, while high errors and deviations showed in the winter. Compared with the former study, low temperatures, high EVI, and wet conditions might cause a rapidly decreasing trend [34]. This study compared the variation from month to month in detail, confirming the situation because the seasons were classified according to temperature.

Compared to the frequency used in generating products, the performance and applicability were comprehensible. SMAP involved an L-band radiometer and a C-band radar, which could penetrate the soil effectively [28]. The AMSR2 products mainly relied on X- and C-band radiometers, which could not penetrate as deep as L-band observations. In addition, radio frequency interference issues were observed at the C-band that influenced the observation [37]. The AMSR2 products were more affected by vegetation status, related to a relatively poor performance. The differences between months and seasons also can be explained because the vegetation status varies with time. The ESA CCI products harmonized the ACTIVE and PASSIVE datasets, performing better in both densely and sparsely vegetated areas, which is mentioned in Section 2.1.

### 4.3. Applicability for Evaluation of Soil Moisture Products

The applicability may change slightly when more factors are considered. An in-depth mechanism analysis is needed to explain the reasons for the differences in land cover types of the five products, especially the poor performance over forests. Soil properties may be connected to the results, and soil types will be considered. The factors that may influence the geospatial evaluation will be taken into consideration in further study, such as climate types and soil texture. In addition, the comparison of different data fusions will be considered because the complementarity between SMAP and ESA CCI was further demonstrated under different climate conditions [37,38].

When we assessed the impact of the ISMN networks, the MCD12Q1 product was used to obtain the land cover types. However, the spatial resolution limited the accurate types of the different stations, resulting in a change of land cover types shown in the MCD12Q1 product. For the observation networks, the land cover proportion will also influence the evaluation. The dominant land cover types over the ARM, FLUXNET-AMERIFLUX and the PBO_H2O networks were obvious, which occupied more than 80% of the land cover types for each network; thus, the performance of the networks could be related to the land cover types. The impact of the land cover types over other networks will be split and assessed in the future.

The evaluation based on the MCD12Q1 product was related to the distribution of the networks. The networks distributed over the whole continental US (e.g., USCRN) could be applied to the whole area, while others, such as ARM and iRON, could not. Future assessments over the land cover types will try to reduce the impact of these differences.

## 5. Conclusions

Satellite-based methods provide plenty of soil moisture products, and evaluations can better realize soil moisture monitoring in different areas. Based on the assessment results, a possible development of soil moisture products can be considered. It is necessary to continue carrying on the evaluation from more aspects.

This study evaluated the performance of five soil moisture products in the continental US and found that five products generally overestimated ISMN data. According to the overall performance, R increased slightly from the annual to seasonal to monthly scales, while ubRMSE increased at the same time, which meant a correlation with errors. The results at the different months and seasons show the impacts of seasonal variation, especially for the AMSR2 products. A lower correlation in the summer might be affected by the vegetation status and higher errors, and deviations in the winter might be related to the low temperatures. The AMSR2 products were supposed to be optimized because of the increasing deviations and errors shown from 2016 to 2020.

The evaluation based on the observation networks showed the quality and accessibility, considering the distribution of the networks. ESA CCI tended to perform better over RMSE, ubRMAE, and MAE with lower errors less than 0.1 m^3^/m^3^. Over the FLUXNET-AMERIFLUX networks, the AMSR_AVG product performed better than both ascending and descending products. The PBO_H2O and USCRN networks not only distributed uniformly, but also performed well and were suitable for reflecting the distribution characteristics of soil moisture in the continental US. In addition to the networks performing well that were located in central California, the geographical environment and climate conditions might take effect.

Land cover types also impact the evaluation. Grasslands and shrublands were the main type that had most stations and occupied 80% over the networks except for FLUXNET-AMERIFLUX and iRON; a better performance also showed over this type and indicated applicability. Savannas were the second major type of stations. Poor performance showed over forests, where deviations were obvious. ESA CCI performed better over land cover types, while SMAP showed no correlation with significant deviations and errors over forests. The geographic distribution was closely related to the performance of the networks and the composition of the land cover types.

In terms of data quality control, SMAP was the closest to ISMN data from all aspects of the statistical characteristics. The smallest deviation from ISMN data reflected the effective control of the differences, but the correlation needs to be improved. Like SMAP in the deviation, ESA CCI performed smoothly at different time scales with better quality control. The AMSR2 products showed fluctuations and more outliers, indicating no better quality.

SMAP is more suitable for scenarios with small deviations from ISMN data and is suitable for evaluation between months. ESA CCI is suitable for most scenarios at different time scales, both in terms of correlations and differences. The performance of the AMSR2 products varied greatly especially at different months, and a poor correlation was often accompanied by a good performance of differences. Its performance should be seriously considered before application.

## Figures and Tables

**Table 1:** Information about products.

Products	Active or Passive	Frequency	Generating Methods
SMAP	Active–Passive	L-band radiometer and C-band radar	Baseline SMAP-Sentinel1 Active–Passive algorithm
AMSR2	Active	C- and X-band radiometers	LPRM algorithm
ESA CCI	Active–Passive	/	ESA CCI SM Merging algorithm

**Table 2:** Number of stations with different land cover types over observation networks.

Network	Forests	Grasslands and Shrublands	Savannas	Barren and Sparse Vegetation	Others
ARM	0	16	1	0	0
FLUXNET-AMERIFLUX	0	0	0	0	1
PBO_H2O	2	113	5	14	1
SOILSCAPE	0	53	35	0	0
USCRN	10	66	30	5	1
iRON	0	3	5	1	0

**Table 3:** PBIAS of five products over different observation networks.

Network	SMAP	ESA CCI	AMSR_AVG	AMSR_AS	AMSR_DE
ARM	0.0816	−0.0894	−0.2114	−0.1096	−0.3132
FLUXNET-AMERIFLUX	/	/	−0.2940	−0.3176	−0.2703
PBO_H2O	0.1110	−0.3201	−0.1321	−0.0001	−0.2642
SOILSCAPE	−0.3245	−0.3423	−0.3405	−0.2212	−0.4599
USCRN	−0.2514	−0.2492	−0.4851	−0.2843	−0.6860
iRON	0.2756	−0.1022	−0.5437	−0.8389	−0.2485

**Table 4:** Products with the best performance over different observation networks.

Network	R	RMSE (m^3^/m^3^)	ubRMSE (m^3^/m^3^)	MAE (m^3^/m^3^)	MBE (m^3^/m^3^)	Num1 ^1^	Num2 ^2^
ARM	ESA CCI 0.5110 *	ESA CCI 0.0712	ESA CCI 0.0684	ESA CCI 0.0548	SMAP 0.0180	17	882
FLUXNET-AMERIFLUX	AMSR_AVG 0.6245 **	AMSR_AVG 0.0985	AMSR_AVG 0.0496	AMSR_AVG 0.0851	AMSR_DE −0.0783	1	19
PBO_H2O	AMSR_AVG 0.6431 *	ESA CCI 0.0651	ESA CCI 0.0537	SMAP 0.0471	AMSR_DE 0.0000	135	1561
SOILSCAPE	AMSR_AVG 0.6707 *	ESA CCI 0.0937	ESA CCI 0.0777	ESA CCI 0.0788	SMAP −0.0514	88	541
USCRN	ESA CCI 0.7489 *	ESA CCI 0.0787	ESA CCI 0.0664	ESA CCI 0.0654	SMAP −0.0413	112	4720
iRON	ESA CCI 0.4479 *	ESA CCI 0.0603	ESA CCI 0.0588	ESA CCI 0.0475	ESA CCI −0.0135	9	180

^1^ Num1: Number of observation stations. ^2^ Num2: Number of data corresponding to observation station. *: *p*-value < 0.0001and **: *p*-value < 0.005.

**Table 5:** Statistics of five products.

	Normal Point Statistics (units: m^3^/m^3^)						Number of Points	
	Mean	Minimum	Lower Quartile	Median	Upper Quartile	Maximum	Normal Point	Outlier Point
ISMN	0.1622	0.0017	0.0838	0.1466	0.2310	0.4438	8034	0
SMAP	0.1774	0.0200	0.0873	0.1488	0.2527	0.4980	5743	103
ESA CCI	0.2007	0.0201	0.1381	0.1994	0.2611	0.3990	7345	0
AMSR_AVG	0.2074	0.0000	0.1100	0.1950	0.3000	0.5850	7430	257
AMSR_AS	0.1862	0.0000	0.0900	0.1700	0.2800	0.5600	7493	194
AMSR_DE	0.2275	0.0000	0.1200	0.2200	0.3300	0.6400	7376	311

## Data Availability

ISMN data are available at https://ismn.geo.tuwien.ac.at/ (accessed on 15 July 2022), ESA CCI data are available at https://www.esa-soilmoisture-cci.org/ (accessed on 15 July 2022), AMSR2 and SMAP data are available at https://search.earthdata.nasa.gov/search (accessed on 15 July 2022).

## Abbreviations

**Abbreviations**	**Name**
AMSR2	Advanced Microwave Scanning Radiometer 2
AMSR_AVG	AMSR2 average product
AMSR_AS	AMSR2 ascending product
AMSR_DE	AMSR2 descending product
CCI	Climate Change Initiative
ESA	European Space Agency
GPS	Global Positioning System
ISMN	International Soil Moisture Network
LPRM	Land Parameter Retrieval Model
NASA	National Aeronautics and Space Administration
SMAP	Soil Moisture Active Passive
**Indicators**	
PBIAS	Percentage of BIAS
R	Pearson Correlation Coefficient
RMSE	Root Mean Square Error
ubRMSE	unbiased Root Mean Square Error
MAE	Mean Absolute Error
MBE	Mean Bias Error
**Observation networks**	
ARM	Atmospheric Radiation Measurement network
FLUXNET-AMERIFLUX	AmeriFlux FLUXNET
PBO_H2O	PBO_H2O network
SOILSCAPE	Soil Moisture Sensing Controller And oPtimal Estimator
USCRN	U.S. Climate Reference Network
iRON	Interactive Roaring Fork Observation Network

## Appendix A

**Table A1:** Table of classification comparison.

Classification Used in the Study	IGBP Classification from MCD12Q1
forests	Evergreen Needleleaf Forests
	Evergreen Broadleaf Forests
	Deciduous Broadleaf Forests
	Mixed Forests
grasslands and shrublands	Closed Shrublands
	Open Shrublands
	Grasslands
	Croplands
	Cropland/Natural Vegetation Mo-saics
savannas	Woody Savannas
	Savannas
barren and sparse vegetation	Urban and Built-up Lands
	Barren
